# The Role of Oxidative Stress in the Aging Heart

**DOI:** 10.3390/antiox11020336

**Published:** 2022-02-09

**Authors:** Luana U Pagan, Mariana J Gomes, Mariana Gatto, Gustavo A F Mota, Katashi Okoshi, Marina P Okoshi

**Affiliations:** 1 Internal Medicine Department, Botucatu Medical School, Sao Paulo State University (UNESP), Botucatu 18618 687, Brazil; luana.pagan@unesp.br (L.U.P.); mariana.gatto@unesp.br (M.G.); gustavo.mota@unesp.br (G.A.F.M.); katashi.okoshi@unesp.br (K.O.); 2 Brigham and Women’s Hospital, Harvard Medical School, Boston, MA 02115, USA; mgomes18@bwh.harvard.edu

**Keywords:** senescence, cardiac remodeling, reactive oxygen species, cardiac metabolism, aged heart

## Abstract

Medical advances and the availability of diagnostic tools have considerably increased life expectancy and, consequently, the elderly segment of the world population. As age is a major risk factor in cardiovascular disease (CVD), it is critical to understand the changes in cardiac structure and function during the aging process. The phenotypes and molecular mechanisms of cardiac aging include several factors. An increase in oxidative stress is a major player in cardiac aging. Reactive oxygen species (ROS) production is an important mechanism for maintaining physiological processes; its generation is regulated by a system of antioxidant enzymes. Oxidative stress occurs from an imbalance between ROS production and antioxidant defenses resulting in the accumulation of free radicals. In the heart, ROS activate signaling pathways involved in myocyte hypertrophy, interstitial fibrosis, contractile dysfunction, and inflammation thereby affecting cell structure and function, and contributing to cardiac damage and remodeling. In this manuscript, we review recent published research on cardiac aging. We summarize the aging heart biology, highlighting key molecular pathways and cellular processes that underlie the redox signaling changes during aging. Main ROS sources, antioxidant defenses, and the role of dysfunctional mitochondria in the aging heart are addressed. As metabolism changes contribute to cardiac aging, we also comment on the most prevalent metabolic alterations. This review will help us to understand the mechanisms involved in the heart aging process and will provide a background for attractive molecular targets to prevent age-driven pathology of the heart. A greater understanding of the processes involved in cardiac aging may facilitate our ability to mitigate the escalating burden of CVD in older individuals and promote healthy cardiac aging.

## 1. Introduction

Medical advances and the availability of diagnostic tools have considerably increased life expectancy and, consequently, the elderly segment of the world population [1]. The number of people aged 60 years and over worldwide is expected to increase from 841 million in 2013 to approximately 2 billion in 2050 [2].

Aging is characterized by complex biological changes [3]. As aging is hard to define, populational studies have used chronological definitions [4]. However, biological aging is determined by a combination of characteristics, such as genetic load, lifestyle, risk factors for cardiovascular disease, comorbidities, psychological condition, social and economic background, functional capacity, biological stress exposure, and homeostatic capacity changes [3,5,6]. Therefore, although the chronological years are unchangeable, other factors that modulate aging may be modified [3,7]. As age is a major risk factor for cardiovascular disease (CVD), it is critical to understand the cardiac changes that occur during the aging process [8]. Advances in cardiovascular research have identified increased oxidative stress as a major pathophysiological mechanism in the development and progression of cardiac alterations during aging [9]. Here, we summarize aging heart biology, highlighting key molecular pathways and cellular processes that underlie the redox signaling changes. Main reactive oxygen species (ROS) sources, the antioxidant defenses, and the role of dysfunctional mitochondria in the aging heart are discussed. As metabolism changes contribute to cardiac aging, we also comment on the most prevalent metabolic alterations.

## 2. Aging Cardiac Changes

Several molecular, biochemical, structural, and functional changes have been observed in the heart of apparently healthy individuals [10]. Although the changes are commonly characterized as “normal”, some alterations resemble the early stages of diseases such as systemic arterial hypertension and coronary atherosclerosis. Thus, cardiovascular aging and cardiovascular disease are connected and present molecular links that have not been completely established [3].

Among structural myocardial alterations, a decrease in the number of myocytes and in the left ventricular (LV) end-diastolic dimension is highlighted [11,12]. A reduction in myocyte number is mainly caused by apoptosis [12]. As there is a size increase in the remaining myocytes, increased LV wall thickness is often observed [13]. When LV mass is preserved, the combination of reduced LV end-diastolic dimension with increased LV wall thickness is known as concentric remodeling [13]. The Framingham Heart Study [14] showed that the LV mass may increase with advancing aging; however, the study was primarily carried out in individuals with an increased burden of risk factors for CVD, such as diabetes mellitus, systemic arterial hypertension, and higher body mass index. The left atrium is also subjected to aging modifications. The main structural modification is an increase in left atrium diameter and volume [15].

Another commonly found structural change is an increase in collagen tissue which also presents altered physical properties [16]. Fibrillar collagen Types I and III are the predominant forms in the myocardial extracellular matrix. Type I collagen is composed of thick fibers with a high tensile strength, and Type III collagen is formed of small diameter fibers with a low tensile strength [17]. Collagen I/III ratio in the human heart remains minimally evaluated or outdated in the literature. Nonetheless, there appears to be a 4-to-5-fold increase in myocardial collagen I/III ratio over physiological values in aged persons [18]. Cardiovascular magnetic resonance images have shown that higher diffuse myocardial fibrosis can be found in older individuals [13]. Similar changes in collagen I/III ratio occurs in vessels during aging and collaborates to increase arterial stiffness [18,19].

The most evident functional alteration is related to LV diastolic function [20,21]. Increased interstitial collagen increases myocardial stiffness and reduces ventricular compliance [22,23]. As a consequence, a progressive reduction in mitral early-diastolic inflow peak velocity (E wave) can be observed [22]. Interestingly, this index starts to reduce from the age of 20 years and by the age of 80 its value is approximately 50% lower than at 20 years of age [22]. Changed LV diastolic properties may lead to diastolic dysfunction and heart failure with preserved ejection fraction which is more common in the elderly [8]. From 45 to 95 years of age, overall lifetime risk for heart failure ranges from 20% to 45% [8]. It has not currently been established whether diastolic dysfunction is part natural of the aging process or is caused or influenced by pathological and lifestyle conditions such as smoking, sedentarism, and nutritional imbalance [3,24,25].

Some experimental studies have suggested that contractile function is also impaired with age [26,27]. Cardiac cycle duration is prolonged with an increase in both contraction and relaxation times. Human studies have also shown a worsening in systolic function. Advancing age has revealed a decline in myocardial and LV longitudinal deformation rates [28], impaired LV dyssynchrony [29], and abnormal mitral annular plane systolic excursion [30]. 

Left atrium function is composed of three components: reservoir, conduction and pump. During aging, as atrial compliance during ventricular systole tends to reduce, the reservoir function is impaired [31]. The pump function, also called active atrial contraction, is not fully understood and studies have shown both improved and impaired function over the years [32,33]. When LV compliance is reduced, left atrium active emptying fraction increases and passive emptying fraction decreases [34]. 

Several genetic, molecular and biochemical abnormalities are involved in the structural and functional myocardial changes during aging [9,26,35,36,37]. These abnormalities are mainly characterized by changes in contractile proteins and cardiac excitation-contraction coupling, decreased contractile response to β-adrenergic stimulation, increased systemic and myocardial oxidative stress and renin–angiotensin activity, and metabolic changes [3,26,27].

## 3. Energetic Metabolism and Cardiac Aging

The cardiac muscle consists of aerobic tissue with high energy demand and is dependent on a large amount of adenosine triphosphate (ATP) which comes mostly from the oxidation of fatty acids, carbohydrates, ketone bodies, and amino acids [38]. In the healthy heart, approximately 60% of synthesized energy comes from the oxidation of fatty acids and almost 40% from the oxidation of glucose and lactate; ketone bodies and amino acids only have a minor contribution [39]. The role of each substrate can be altered depending on changes in substrate availability, organ demand, feeding status, and the presence of ischemia or hypoxia [40].

Aging causes a gradual loss of cellular homeostasis [41]. These changes lead to metabolic alterations in the heart characterized by decreased oxidation of fatty acids and increased glucose utilization in combination with changed expression of genes encoding enzymes participants in the cardiac metabolism [42,43]. During aging, there is a decline in respiratory proteins interfering with the use of fatty acids. On the other hand, there is an increase in glycolysis-related proteins [44]. This process has been defined as a remodeling of energy metabolism, which can also be observed in some heart diseases [42,45].

An imbalance between absorption and oxidation of fatty acids in aging hearts leads to the accumulation of fatty acids that are incorporated into triglycerides, phospholipids and other lipid subspecies [46]. In the elderly myocardium, lipid accumulation with increased levels of lipid transporter suggests an inability to oxidize rather than to transport the lipids [47,48]. Thus, the combination of increased lipid uptake and reduced oxidation increases the amount of lipids in the cardiac tissue that may be responsible for lipotoxicity and the onset of cardiac diseases.

Glucose is delivered to cardiomyocytes from the bloodstream or intracellular glycogen storage [42]. Increased energy production by glycolysis during changes in substrate availability or pathological changes may be beneficial as it ensures the supply of ATP to the contractile function [49,50]. In the aged heart, greater glycolysis was found compared to fatty acid utilization [51]. This compensatory mechanism can balance energy deficiency. However, it is not clear whether this mechanism is advantageous in the long term. In cardiac hypertrophy there is a decreased fatty acid oxidation with increased glucose utilization [52], which has been considered an adaptive compensatory response [53,54,55]. On the other hand, increased glucose uptake during hyperglycemia was harmful to cardiac myocytes [56]. Finally, in hearts from transgenic mice, increased glucose uptake improved diastolic function, preserved myocardial energy, and attenuated susceptibility to ischemia-reperfusion caused by aging [57]. Although suggesting that the heart can adapt to a long-term increase in intracellular glucose with no adverse effects, this issue needs a better clarification. 

Recent studies have focused on ketone bodies as an energy source for cardiac muscle [58,59,60]. In addition to being an efficient substrate for cardiac metabolism as it requires less oxygen per produced ATP [61], the oxidation of ketone bodies has the potential to alleviate age-related cardiovascular complications [38]. Its energy-saving properties improve cardiac metabolic efficiency by increasing ATP production [58]. The oxidation of ketones in the myocardium is proportional to their plasma concentrations; an increase in ketones levels could have cardioprotective effects by enhancing cardiac function and reducing cardiac inflammation [62]. The production of ketone bodies in the liver is higher in elderly mice than their young counterparts [63]. Enzymatic alterations involved in ketone body metabolism have been suggested as a mechanism for the age-related metabolic shift towards increased consumption of ketone bodies and as an alternative source of energy supply [64]. Several studies have shown the antioxidant effects of ketone bodies in elderly hearts, with improved mitochondrial repair mechanisms and increased ratio of reduced-to-oxidized glutathione [65,66,67].

One of the enzymes responsible for cell energy homeostasis is adenosine monophosphate activated protein kinase (AMPK), which also regulates mitochondrial production of ROS [68,69]. Activation of AMPK modulates several biochemical events, including glucose uptake, glycolysis, fatty acid oxidation, and mitochondrial biogenesis [42,70]. These processes significantly contribute to increasing ATP levels and restoring myocardial contractile efficiency [71]. Aging can impair the AMPK signaling pathway. AMPK activation protects the elderly heart from oxidative stress through activation of nuclear factor erythroid 2-related factor 2 and serine/threonine-protein kinase 2 [72,73,74,75]. Recent studies have shown that hormones and natural substances may activate AMPK, inhibiting ROS production and preserving cardiomyocytes [73,76,77,78]. These findings suggest that AMPK protects the heart against oxidative stress and acts as a potential therapeutic target.

Alterations in cardiac substrate use have been associated with CVD [79,80]. Changes in substrate preference from fatty acid to glucose and ketone bodies were described in the hypertrophied or failing heart [60,81,82]. The decrease in fat oxidation is associated with reduced expression of genes involved in fatty acid uptake and mitochondrial β-oxidation [80]. In the pathological remodeling caused by diabetes, greater utilization of fatty acid and ketone bodies was observed and assigned to the increased fatty acid supply and insulin resistance [79]. In the ischemic disease, cessation of oxidative phosphorylation leads to the use of alternative pathways [80]. High-energy phosphates in the form of creatine phosphate are used, but they are quickly depleted, and anaerobic glycolysis becomes the main ATP source [83]. If ischemia continues, the rate of glycolysis decreases, lactate accumulates, and acidosis inhibits several enzymes of the glycolytic pathway. The ATP reduction in the ischemic myocardium is associated with irreversible changes in cardiomyocytes [84].

The contribution of oxidative stress to the metabolic remodeling is not well established. In aging, mitochondrial dysfunction impairs oxidative phosphorylation reducing ATP production and increasing cardiomyocyte ROS [85]. Increased ROS further induces mitochondrial electron transport chain dysfunction leading to a vicious cycle between damaged mitochondria and increased ROS production (see below) [86]. Increased ROS can affect the availability of heart substrates reducing ATP turnover and causing both metabolic remodeling and contractile dysfunction. 

## 4. Oxidative Stress and Aging

ROS were generally considered to be detrimental by-products of cellular metabolism and thought to cause toxic effects associated with several pathological conditions. However, it is now well established that besides playing a role in molecular damage, ROS fulfil second messenger-like functions [87]. A fine balance is necessary for ROS generation to limit cell injury and extend lifespan. For example, moderate ROS levels may increase the tolerance against metabolic, mechanical, and oxidative stressors [88]. Brief periods of increased oxidative stress during ischemia-reperfusion may limit later cellular injury through several pathways such as those involving the mechanistic target of rapamycin (mTOR) or Wnt signaling. However, at increased levels, ROS can damage mitochondria, organelles, and DNA culminating in cell aging and cell demise [89].

Known as redox regulation, the balance between ROS generation and elimination is maintained by complex mechanisms. Dysfunction in any of these mechanisms can disrupt redox homeostasis and increase oxidative stress [90]. Oxidative stress is a major contributor to several age-associated cardiovascular diseases including atherosclerosis [27,91,92]. Gene expression coding for proteins involved in ROS production and clearance pathways are changed with aging in both human and rat hearts leading to increased systemic and cardiac oxidative stress and predisposing to cardiovascular diseases [9,93]. The responsiveness of the aged heart to stress is altered with a decrease in antioxidant capacity along with increased oxidant production [94]. The main ROS source during aging is mitochondria (discussed later), where ROS are mainly generated as a by-product of oxidative phosphorylation [95]. In this section, we will address major endogenous ROS sources including the NADPH oxidase (NOX) family, nitric oxide oxidase, and endoplasmic reticulum.

### 4.1. Sources of ROS Other Than Mitochondria

#### 4.1.1. NADPH Oxidase

Despite the multiple ROS sources, a major source of ROS involved in myocardial redox signalling is the family of NADPH oxidases. Currently, there are seven known proteins in the NOX family—NOX1, NOX2, NOX3, NOX4, NOX5, dual oxidase (DUOX) 1, and DUOX2. All members of the NOX family can drive the NADPH-dependent reduction of O_2_ to O_2_^•−^. NOX4, DUOX1, and DUOX2 may also generate H_2_O_2_ [96]. 

NOX2 consists of a multicomponent complex involving transmembrane flavocytochrome b558—which is the heterodimeric assembly of NOX2 and p22phox—supported by cytosolic protein factors p47phox, p67phox and p40phox, and small GTP-binding proteins (G proteins Rac1 or Rac2). NOX2 activation requires assembly of the multiprotein complex. In brief, NOX2 activation requires phosphorylation of p47phox to induce a conformational change to unmask p47phox’s autoinhibited tandem SH3 domain. Release of the tandem SH3 enables p47phox translocation and binding to the PRR region of p22phox. This interaction provides a scaffold for p67phox and p40phox assembly in the complex. When joined by Rac, this complex enables electron transfer and superoxide production in the presence of NADPH [97].

NOX1 and NOX3 are the closest isoforms to NOX2 and their activation requires cytosolic factors NOXO1 and NOXA1, respectively, homologous to the p47phox and p67phox NOX2 subunits. Unlike other isoforms, NOX4 is constitutively active. NOX5 is endowed with other specificities compared to other NOX isoforms, such as a Ca^2+^-dependent activation. Binding of Ca^2+^ to NOX5’s extra EF-hand domain results in a conformational change which exposes hydrophobic regions that bind to the catalytic core to activate electron transfer [97].

The DUOXes are sequestered in an inactive state in the endoplasmic reticulum. They require a maturation factor (DUOXA1 or DUOXA2) to adopt a conformation consistent with the acquisition of post-translational modifications responsible for the migration of the complex to the plasma membrane. In the presence of DUOXA2, DUOX1 produces O_2_^•−^ while DUOX2 also produces H_2_O_2_ [97].

ROS generated by NOX have been implicated in the pathophysiology of systemic arterial hypertension, atherosclerosis, angiogenesis, and in endothelial dysfunction associated with hypercholesterolemia, diabetes and aging [98]. NOX in cardiovascular cells continuously generates ROS at a low level even in the absence of cell stimulation. NOX activity may be enhanced by several stimuli, such as cyclic stretch, angiotensin II, α-adrenergic agonists, endothelin-1, and TNF-α, many of which are relevant to left ventricular hypertrophy and heart failure [98]. Studies have shown both increased and unchanged myocardial and skeletal muscle NOX activity during cardiac injury [99,100,101,102,103,104,105,106,107]. Differences in experimental models and cardiac injury levels could be involved in the divergent results. NOX2 can play an essential role in age-associated cardiac remodeling by enhancing matrix metallopeptidase activation and the expression of pro-fibrotic factors such as connective tissue growth factor and transforming growth factor-β, and the induction of cardiomyocyte hypertrophy [108].

#### 4.1.2. Nitric Oxide Oxidase

Nitric oxide (NO) is synthesized by NO synthase (NOS) during the catalysis of L-arginine into L-citrulline. Three NOS isoforms have been reported: neuronal NOS (nNOS and NOS1)—linked to intracellular signaling, inducible NOS (iNOS and NOS2)—activated in response to endotoxins or cytokine signals, and endothelial NOS (eNOS and NOS3)—related to vasodilation and vascular regulation. There is high expression of nNOS in neuronal cells, including those in the brain and skeletal and cardiac muscle. NO modulates cardiac function and also has a protective role in the ischemic and failing heart [109]. Cytotoxicity attributed to NO is, to a certain extent, due to peroxynitrite. NOS uncoupling in various diseases results in a dysregulated NO response whereby NO combines with O_2_^−^ to produce peroxynitrite, which interacts with lipids, DNA, and proteins leading to oxidative damage [110].

Changes in NOS or NO concentration are involved in the pathophysiology of aging. NO regulates xanthine oxidase, a ROS source in the heart [111]. Under physiologic conditions, administration of a specific NOS inhibitor induces cardiac mechanoenergetic uncoupling due to an increase in oxygen consumption in relation to the work performed, which was attributed to the ROS production by xanthine oxidase [112]. ROS production by xanthine oxidase is increased in the absence of NOS1 leading to deleterious effects on myocardial excitation–contraction coupling [113]. Deficiency of NOS1 or NOS3 isoforms is associated with cardiac hypertrophy in mice [111]. Loss of both isoforms produces a classic cardiovascular phenotype of aging with concentric left ventricular remodeling [114]. During aging and cardiovascular disease, animal and human studies confirm that NO is dysregulated at various levels, with a decrease in production, tissue half-life, and potency [115].

#### 4.1.3. Endoplasmic Reticulum

The endoplasmic reticulum (ER) regulates calcium homeostasis, lipid metabolism, protein synthesis, and posttranslational modification and trafficking. The ER contains high Ca^2+^ concentration, which is maintained by the active transport of the sarcoendoplasmic reticulum (SR) calcium transport ATPase. Homeostasis in the endoplasmic reticulum may be disrupted by a number of insults, including alterations in intracellular Ca^2+^ transient or redox status [116]. 

The ER is the site of folding secreted proteins. Cellular stress, such as an increase in secretory load or the presence of mutated proteins that cannot be properly folded, can unbalance the relationship between demand for protein folding and ER capacity for protein folding, inducing ER stress [117]. ER senses and responds to stress through signal transduction pathways known as the unfolded protein response [118]. Protein-folding is highly sensitive to endoplasmic reticulum redox status; dysregulation of disulfide bond formation in response to endoplasmic reticulum stress increases luminal oxidative stress and reduces endoplasmic reticulum function [119]. The efficiency of the ER stress recognition system and unfolded protein response signaling declines during aging [120]. Increased ER stress was observed in senescent cardiomyocytes with impaired contractility, which was associated with increased oxidative stress and endoplasmic reticulum stress [120,121]. 

#### 4.1.4. Other ROS Sources

Other endogenous ROS sources include cytochrome P450 and peroxisomal oxidative metabolism [96]. ROS are also produced by exogenous agents, such as chemotherapy drugs, radiation, heavy metals, atmospheric pollutants, chemicals, drugs and xenobiotics [96].

### 4.2. Antioxidant Defense Mechanisms

The heart is equipped with antioxidant systems to scavenge excess ROS [90]. An integrative system of antioxidant enzymes maintains reactive species in a range that promotes physiological cell signaling and minimizes oxidative damage. The primary antioxidant enzymes are superoxide dismutase, catalase, and glutathione peroxidase. Briefly, O_2_ is converted by superoxide dismutase to H_2_O_2_, which is decomposed to water and oxygen by catalase, preventing hydroxyl radical production. Additionally, glutathione peroxidase converts peroxides and hydroxyl radicals into nontoxic forms by oxidation of reduced glutathione into glutathione disulfide and then reduced to glutathione by glutathione reductase [122]. 

Thioredoxins, peroxiredoxins, glutathione peroxidases, p38 mitogen-activated protein kinase (MAPK), sirtuins, and non-enzymatic antioxidants such as glutathione, Vitamin C, Vitamin E and polyphenols also directly act on oxidative agents [96]. Sirtuin 1 is a longevity gene considered to play an important role in cardiovascular aging. Sirtuin 1 expression declines with age. Aging impairs sirtuin 1 activation during ischemic stress increasing the susceptibility of the heart to ischemia/reperfusion injury [123].

### 4.3. ROS-Mediated Signaling

Considering the importance of switch ability between ON and OFF states in intracellular signaling, proteins that can be sensitively and reversibly oxidized by ROS are candidates for mediating the ROS signaling function. Among the 20 amino acids that make up proteins, cysteine is of particular interest, because the thiol moiety in the cysteine side chain is very sensitive to oxidation and can form disulfide bonds with another thiol moiety. Disulfide bonds can be reduced back to the free thiol moiety under physiological intracellular conditions. Therefore, the cysteine residues that exist on the protein surface are considered to be the physiological targets for ROS. This oxidative reaction appears to occur non-specifically, but studies revealed the presence of highly reactive cysteine selectively oxidized by ROS [124].

The central mechanism underlying most ROS-dependent signaling is thought to be the covalent modification of specific cysteine residues found within redox-sensitive target proteins. Oxidation of these reactive cysteine residues can lead to reversible modification and activate or inactivate their target proteins [125]. Examples of transcriptional regulation that uses cysteine oxidation to sense and respond to increased ROS levels include intracellular targets such as Kelch-like ECH associated protein 1 (Keap1), nuclear factor erythroid 2-related factor 2 (Nrf2), and forkhead box O [126]. Expression of Nrf2 is decreased in elderly patients. Strategies to stimulate Nrf2 and enhance endogenous antioxidants may decrease the severity of cardiovascular disease in the elderly [94].

Nuclear transcription factor κB (NF-κB) is another example of redox-dependent activation. Its active form is a heterodimer consisting of 50- and 65-kDa subunits. The heterodimer remains bound to NF-κB inhibitor (IκB) in the cytoplasm. In response to oxidative stress, IκB is phosphorylated, ubiquitinated, and degraded, which unmasks the DNA binding activity of the heterodimer and allows it to translocate to the nucleus where it can activate gene transcription. Depending on the context, ROS can both activate and inhibit NF-κB signaling [127]. 

The sirtuin family of seven enzymes has also been linked to several antioxidant and oxidative stress related processes, including longevity, mitochondrial function, DNA damage repair, and metabolism [128]. Sirtuin 1 was shown to protect the aging heart by inhibiting the endoplasmic reticulum-mediated apoptosis [120]. Finally, a major pathophysiological characteristic of aging involves the elevated and sustained endogenous expression of the stress response signaling pathways that involve p38 MAPK, JNK, and NF-κB [92]. 

Mitochondria can intensify ROS production by a phenomenon called ROS-induced ROS release, which occurs when ROS produced in an intracellular niche triggers ROS formation at other cell sites [129,130]. The increase in ROS synthesis can occur through ion channels of the mitochondrial inner membrane such as mitochondrial permeability transition pore and ATP-dependent K-channel, which, once activated, increases NADPH consumption and H_2_O_2_ production forming a vicious cycle [131,132].

## 5. Aging Mitochondria

One fundamental mechanism in increased oxidative stress during aging is the dysregulation of mitochondrial dynamics and quality control [9,27]. Cardiomyocytes present a large number of mitochondria which supply the great demand for energy generating ATP. The cardiomyocyte aging process is accompanied by several mitochondrial alterations, such as ultrastructure abnormalities, electron transport defects, genome mutations, mitochondrial biogenesis changes accompanied by clonal expansion of dysfunctional mitochondria, ROS production increase, and mitophagy suppression [133,134].

### 5.1. Respiratory Chain

In the myocardium, the reticular form of mitochondria allows ATP synthesis throughout the entire mitochondrial extension [135]. This organelle has binding structures that favor the communication and distribution of ATP between them; the binding sites tend to be damaged with advancing age [135,136]. Electron microscopy of rat myocardium showed that aging affects the inner mitochondrial membrane, where the respiratory complexes are housed and ATP is synthesized [136].

Mitochondria generate energy in the form of ATP through oxidative phosphorylation of carbohydrates and fatty acids. Energy supply is regulated under different scenarios enabling the heart to have energetic plasticity [137]. The electron transport chain is represented by large enzymatic complexes, numbered from I to IV, which form the respirasomes. Oxygen production comes from electron transfer through the redox potential gradient of nicotinamide adenine dinucleotide (NADH) or flavin adenine dinucleotide H2 (FADH2). The transfer is accomplished by active transport of hydrogen ions between mitochondrial membranes [94]. Briefly, NADH delivers electrons to complex I (NADH-coenzyme Q-reductase) which transfers electrons to complex III (coenzyme QH2-cytocrome c reductase) and complex IV (cytochrome c oxidase). The energy released from the passage of electrons through the three complexes displaces protons out of the mitochondrial matrix forming an electrochemical gradient between the inner membranes of the mitochondria, which results in ATP generation. Complex II participates in the respiratory chain when electrons in FADH2 enter the respiratory chain via coenzyme Q-succinate reductase [94,138]. The mitochondrial ability to generate ATP differs in physiological and pathological conditions; since the heart is highly metabolic, any failure in the respiratory performance can impair its performance [139].

The activity of respiratory complexes III, IV, and V is impaired with aging [94]. Complex III is a major source of free radicals into mitochondria; it catalyzes the transfer of electrons from ubiquinol to cytochrome c in the respiratory chain [140]. Aging modifies the binding locus of mixothiazole in the QO site of cytochrome b [141]. The functional deficiency in the complex III QO site triggers electron leakage increasing ROS production in interfibrillar mitochondria [142].

Alterations in the interactions between mitochondria and ER play fundamental roles in the development and progression of cardiac aging and disease [143,144]. Contraction of myocytes depends on the adequate supply of both ATP and Ca^2+^. Most of the Ca^2+^ needed for contraction is released by the sarcoplasmatic reticulum (SR) and ATP is provided by mitochondria [35]. During the excitation-contraction (EC) coupling, the transverse tubules allow the transduction of the action potential into Ca^2+^ release from the intracellular stores [145]. The function of mitochondria and the Ca^2+^ releasing mechanism are also controlled by their cross-talk: Ca^2+^ released for cell contraction enters mitochondria via the mitochondrial Ca^2+^ uniporter to stimulate the respiratory chain, and the ROS produced by mitochondria modulates SR Ca^2+^ release [145]. Therefore, the increased generation of mitochondrial ROS may change Ca^2+^ handling and vice versa [146]. Communication between ER and mitochondria can be disrupted by the ER stress, which impairs mitochondrial complex I activity and increases ROS production [147,148,149,150,151]. Cardiomyocytes from aged hearts have decreased levels of mitochondrial Ca^2+^ acquisition [152], which is improved by the pharmacological control of ER stress [147,153].

### 5.2. Structural Changes

The heart possesses two types of mitochondria: subsarcolemmal, located below the plasma membrane, and interfibrillar, nestled between the myofibrils. The different mitochondria types allow myocytes to properly respond to different stimuli [154]. The interfibrillar subtype is the most affected by age [155]. Although controversial, it has been reported that both mitochondria types increase in size and the interfibrillar mitochondria lose their cristae during aging. These alterations reduce the extension of the contact surface of inner mitochondrial membrane, which functions as a platform for anchoring the respiratory complexes [156]. Thus, while respiration by subsarcolemmal mitochondria is not affected by aging, interfibrillar mitochondria have decreased oxidative phosphorylation mainly due to defects in the selective donation of complex IV and cytochrome c oxidase [157].

Cardiolipin is a phospholipid widely distributed across the inner membrane of mitochondria. It plays an essential role in maintaining the structural integrity of the cristae and the interaction between respiratory complexes [158]. Studies have suggested that cardiolipin is decreased or structurally changed during senescence [159,160]. Increased oxidative stress contributes to reduced cardiolipin levels [161]. Cardiolipin levels were restored after supplementation with acetyl-L-carnitine, a component of the mitochondrial membrane, in aged rats [162]. Alterations in binding between cardiolipin and cytochrome c oxidase may induce structural modifications that facilitate oxidation of the cytochrome c heme ligand creating a cytochrome with a peroxidase function that oxidizes cardiolipin [163]. Conformational changes decrease the cardiolipin function in the respiratory complexes damaging the inner mitochondrial membranes, facilitating the migration of cytochrome c to the cytosol and inducing pro-apoptotic cell death [161,164].

### 5.3. Mitochondrial DNA and Oxidative Stress

Mitochondria are not only a main cellular source of ROS, but also a major target for ROS [165]. Mitochondrial mutagenesis results from errors during mitochondria DNA (mtDNA) replication [166,167]. Mutations and deletions in mtDNA are more frequent in the hearts of aged than young animals and are related to increased cardiac apoptosis [168]. Somatic mutations in mtDNA are involved in several aging characteristics including mitochondrial dysfunction and reduced ATP production [169,170]. PolyG mice expressing mtDNA polymerase defects harbor mtDNA mutations or deletions in all tissues and show early cardiac aging with cardiac hypertrophy and dysfunction [168,171]. On the other hand, overexpression of catalase targeted to mitochondria (mCAT) prolonged murine lifespan by 17% to 21% and protected mice from cardiac aging, providing direct evidence of the role played of mitochondrial ROS in the aging heart [172,173].

### 5.4. Mitophagy and Cardiac Aging

Autophagy is the process of delivering cellular elements to lysosome degradation. In the heart, inhibition of autophagy results in age-related cardiac disease [72,174]. Autophagy deficient mice present sarcomere disruption, collapsed mitochondria, impaired cardiac function, and reduced survival [175]. Cardiomyocyte autophagy may be modulated by activating autophagic repressors such as Mst1 and inhibiting autophagy activators such as sirtuin 1 [176]. Increased oxidative stress plays an important role in autophagy dysregulation [177].

Damaged mitochondria are eliminated through a specific autophagic process called mitochondrial autophagy or mitophagy [178,179]. Defective mitophagy may trigger several heart disorders [94,177]. In the aging heart, reduced mitophagy was related to accumulation of injured mitochondria, which produce a large amount of ROS. During ischemic damage or cardiac hypertrophy, mitophagy is essential to clear defective mitochondria and avoid oxidative damage [180]. As previously reported, increased oxidative stress contributes to a vicious cycle between the presence of damaged mitochondria and increased ROS production [44,181].

**Figure 1:**
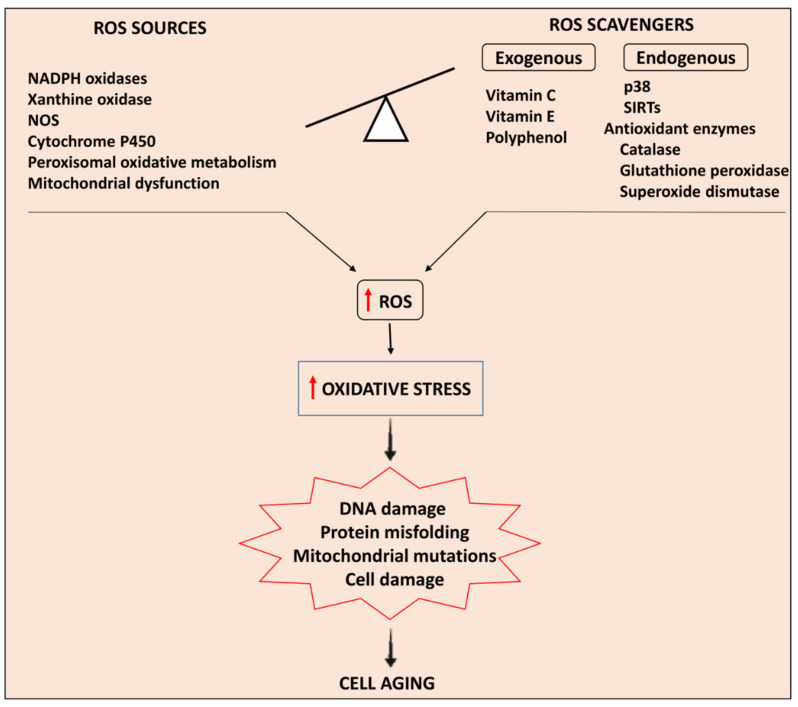
Summary of the mechanisms involved in oxidative stress-induced cardiac myocyte aging. The imbalance between ROS sources and ROS scavengers increases oxidative stress that triggers DNA damage, protein misfolding, mitochondrial mutations, and cell damage culminating in cell aging. NOS: nitric oxide synthase; p38: p38 mitogen activated protein kinase; SIRTs: sirtuins; ROS: reactive oxygen species.

Figure 1 summarizes the imbalance between ROS sources and ROS scavengers that increases oxidative stress and induces cardiac myocyte aging.

**Figure 2:**
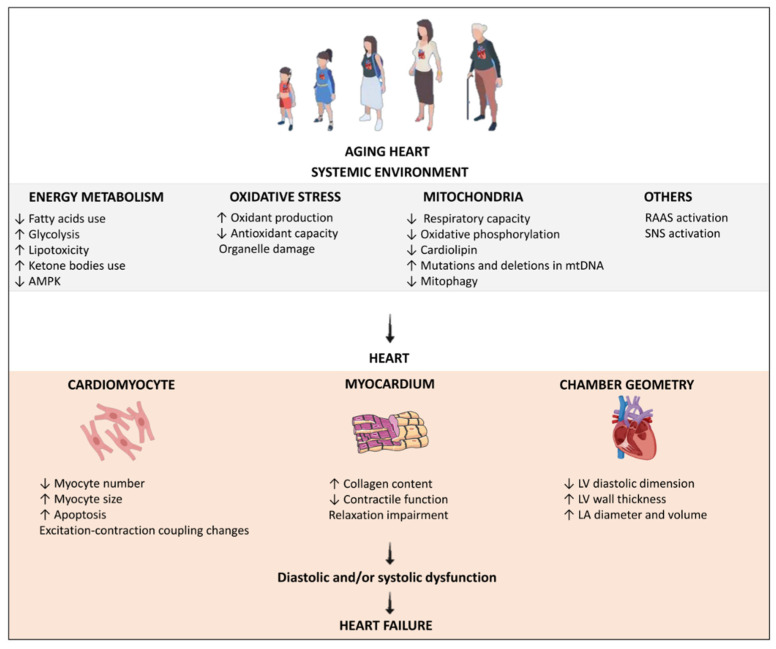
The mechanisms involved in heart structure and function impairment during cardiac aging. AMPK: adenosine monophosphate activated protein kinase; RAAS: renin-angiotensin-aldosterone system; SNS: sympathetic nervous system; LV: left ventricle; LA: left atrium.

Figure 2 shows a summary of the mechanisms involved in heart structure and function impairment during heart aging.

Currently, substantial research has been conducted to better clarify the role of oxidative stress in cardiac aging and to find specific drugs to modulate oxidative stress. Physical exercise has been extensively studied as a non-pharmacologic treatment to reduce systemic and muscular oxidative stress and improve functional capacity under physiological and pathological conditions [88,182,183,184]. Mitochondrial health in cardiomyocytes is associated with extended longevity in rats with higher intrinsic exercise capacity; probably, these findings can be translated to other populations as predictors of health and survival outcomes [185].

## 6. Conclusions

The aging heart is characterized by molecular, biochemical, structural, and functional changes. Excessive oxidative stress and metabolic alterations play a major role in the aging process. The understanding of reactive oxygen species as signaling molecules in several signaling pathways is highly relevant for the development of novel therapies to modulate the production and effects of the reactive oxygen species in aging and related events.
